# Assessment of Ventilation Distribution during Laparoscopic Bariatric Surgery: An Electrical Impedance Tomography Study

**DOI:** 10.1155/2016/7423162

**Published:** 2016-12-12

**Authors:** Michal Stankiewicz-Rudnicki, Wojciech Gaszynski, Tomasz Gaszynski

**Affiliations:** Department of Anaesthesiology and Intensive Therapy, Medical University of Lodz, Lodz, Poland

## Abstract

*Introduction*. The aim of the study was to assess changes of regional ventilation distribution at the level of the 3rd intercostal space in the lungs of morbidly obese patients as a result of general anaesthesia and laparoscopic surgery as well as the relation of these changes to lung mechanics. We also wanted to determine if positive end-expiratory pressure of 10 cm H_2_O prevents the expected atelectasis in the morbidly obese patients during general anaesthesia.* Materials and Methods*. 49 patients completed the examination and were randomized to 2 groups: ventilated without positive end-expiratory pressure (PEEP 0) and with PEEP of 10 cm H_2_O (PEEP 10) preceded by a recruitment maneuver with peak inspiratory pressure of 40 cm H_2_O. Impedance Ratio (IR) was utilized to examine ventilation distribution changes as a result of anaesthesia, pneumoperitoneum, and change of body position. We also analyzed intraoperative respiratory mechanics and pulse oximetry values.* Results.* In both groups general anaesthesia caused a ventilation shift towards the nondependent lungs which was not further intensified after pneumoperitoneum. Reverse Trendelenburg position promoted homogeneous ventilation distribution. Respiratory system compliance was reduced after insufflation and improved after exsufflation of pneumoperitoneum. There were no statistically significant differences in ventilation distribution between the examined groups. Respiratory system compliance, plateau pressure, and pulse oximetry values were higher in PEEP 10.* Conclusions.* Changes of ventilation distribution in the obese do occur at cranial lung regions. During pneumoperitoneum alterations of ventilation distribution may not follow the direction of the changes of lung mechanics. In the obese patients PEEP level of 10 cm H_2_O preceded by a recruitment maneuver improves respiratory compliance and oxygenation but does not eliminate atelectasis induced by general anaesthesia.

## 1. Introduction

General anaesthesia in morbidly obese patients is marked by a significant reduction in functional residual capacity (FRC) and formation of atelectasis in the dependent lung regions, that is, the lowest lung areas in relation to gravity in a particular body position [1]. This is responsible for the increased risk of intraoperative hypoxia and postoperative pulmonary complications in this group of patients [2]. Contemporary bariatric surgery is predominantly laparoscopic and consequently further atelectasis could develop because of raised intraabdominal pressure (IAP) with cranial diaphragm movement [3]. It is therefore recommended that recruitment maneuvers and positive end-expiratory pressure (PEEP) should be used in bariatric anaesthesia [4] to prevent atelectasis and keep the alveoli open in agreement with the “open lung” concept.

Electrical impedance tomography (EIT) enables bedside monitoring of ventilation distribution and its alterations induced by different therapeutic interventions [5], for example, PEEP [6] or change of patient positioning. EIT measures impedance variation of lung tissue using an electrode belt, usually placed between the 5th and 6th intercostal space. The preferred location for EIT monitoring at lower lung regions is due to the fact that they receive most ventilation and impedance variation is expected to correlate better with changes in lung volume [7].

However, EIT monitoring at this level in abdominal surgery may not be feasible [8]. Close proximity to the surgical site and change of patient positioning make access to the electrodes difficult and may preclude the examination. It has been determined to date that at a more cranial level one can also observe changes of ventilation distribution due to different PEEP values [9] but this belt location has not been utilized so far during anaesthesia for laparoscopic procedures.

The purpose of the presented study was to determine if changes of regional ventilation distribution occur at cranial lung regions (3rd intercostal space) during general anaesthesia for laparoscopy in the morbidly obese and to assess them with electrical impedance tomography. Ventilation distribution was examined in two separate groups, ventilated without positive end-expiratory pressure or with PEEP value of 10 cm H_2_O. We also aimed to determine if changes of regional ventilation distribution in the monitored area follow the changes in respiratory system mechanics and if PEEP value of 10 cm H_2_O preceded by a recruitment maneuver prevents the expected atelectasis during general anaesthesia in morbidly obese patients.

## 2. Materials and Methods

The study received approval from the Ethics Committee of the Medical University of Lodz (RNN/583/13/KB). With written informed consent 57 morbidly obese patients aged 18–65 scheduled for laparoscopic gastric banding (LAGB) or laparoscopic sleeve gastrectomy (SG) were recruited for the study. Exclusion criteria were pulmonary comorbidities as well as ischaemic heart disease, heart failure, arrhythmia, and poorly controlled hypertension. Patients were randomly assigned to two different groups, ventilated without positive end-expiratory pressure (group PEEP 0) or with PEEP value of 10 cm H_2_O preceded by a recruitment maneuver with peak inspiratory pressure of 40 cm H_2_O (group PEEP 10). For randomization we used 60 sealed envelopes with group assignment information. We took height and weight measurements and calculated BMI. We also recorded tidal volume settings (VT), the duration of surgery (Ts), and anaesthesia (Ta). Capillary blood gas measurements (CBG) were obtained preoperatively from each patient as is standard procedure in our institution before bariatric surgeries.

Following preoxygenation with FiO_2_ 1,0 for 3 minutes anaesthesia was induced with midazolam 2-3 mg, fentanyl 2 mcg/kg IBW, propofol up to 2 mcg/kg TBW titrated to loss of eyelash reflex, and rocuronium 0,6 mg/kg IBW. Anaesthesia was maintained with sevoflurane administered in low-flow technique with end-tidal (etSevo) concentrations of 0,7–1 MAC and additional fentanyl up to a total dose of 5-6 mcg/kg IBW depending on the type of surgery (LAGB or SG, resp.). Neuromuscular blockade was monitored with TOF technique and sugammadex was given for block reversal upon completion of surgery. After intubation the patients were ventilated with a tidal volume (Vt) of 8 mL/kg IBW with FiO_2_ 0,5 with a PEEP value of either 0 (PEEP 0) or 10 (PEEP 10). Respiratory rate was adjusted to keep end-tidal carbon dioxide (etCO_2_) between 35 and 45 mmHg (Table 3). Additionally in group PEEP 10 before mechanical ventilation was started a recruitment maneuver was performed (two sustained inflations for 10 seconds, each with peak inspiratory pressure of 40 cm H_2_O). During anaesthesia patients were placed in two different positions: ramp position with a bariatric pillow or 40° reverse Trendelenburg.

Static respiratory system compliance (Cresp), plateau pressure (Pplat), etCO_2_ and SpO_2_ values, respiratory rate (RR), mean blood pressure (MBP), and heart rate (HR) were obtained from the anaesthetic workstation (Datex-Ohmeda Aespire or Aestiva). In case SpO_2_ values fell ≤92% during anaesthesia FiO_2_ was increased to 0,8.

In order to monitor regional ventilation distribution we placed a 16 electrode belt of appropriate size selected to match chest circumference at the level of the 3rd intercostal space. To promote high signal quality we applied ultrasound gel between the skin and the EIT belt. The belt was subsequently connected to electrical impedance tomograph (PulmoVista 500, Drager). After high signal quality was confirmed, monitoring was initiated and only interrupted during electrocautery by disconnecting the electrode belt from the impedance tomograph. If signal quality was low measurement was restarted. In case high signal quality was not obtained or was lost during anaesthesia and could not be restored before the next data collection time point, the patient was excluded from the study.

**Figure 1:**
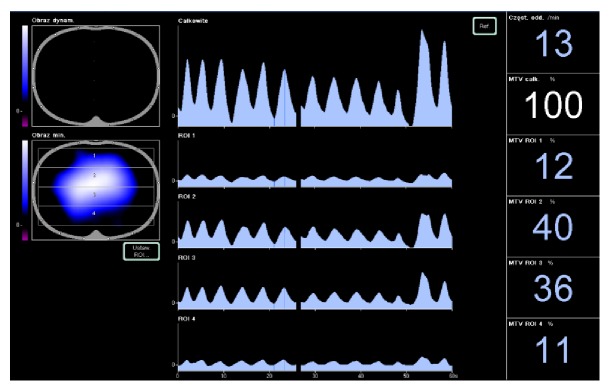
Ventilation distribution in a spontaneously breathing patient (T1). Dependent lungs: ROI 1 and ROI 2; nondependent: ROI 3 and ROI 4. IR = ROI 1 + ROI 2/ROI 3 + ROI 4 = 1,11.

Out of the 16 electrodes one adjacent pair applies a small, imperceptible electrical current to the patient's chest. The remaining ones measure resulting voltages and information about tissue impedance variation is derived. Since location of the current-generating electrodes changes around the thorax a cross-section image of impedance variation is formed analogous to a CT scan. Data on impedance variation are coded with colour. Higher values of impedance variation are associated with better lung ventilation and marked blue. White colour signifies worse ventilation and unventilated area is marked black (Figure 1).

The monitored area is then analyzed by dividing it into 4 layers in a ventrodorsal orientation (Regions of Interest: ROIs) and adjusting their size and position according to the location of the lungs within the thorax. With the patients lying on their back two upper (ROI 1, 2) and two lower (ROI 3, 4) layers are considered images of nondependent and dependent lung regions, respectively (Figure 1).

Data on ventilation distribution were collected as percentage of the total increase in lung impedance variation per each consecutive layer (ROI 1–4) as computed by PulmoVista 500.

**Figure 2:**
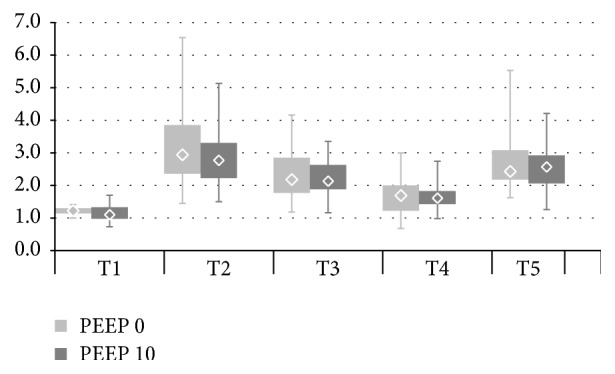
IR ratio (regional ventilation distribution) in groups PEEP 0 and PEEP 10 at T1–T5. PEEP 0: T1 versus T2, T2 versus T3, T2 versus T4, T2 versus T5, T3 versus T4, and T4 versus T5; all* p *< 0,002. PEEP 10: T1 versus T2, T2 versus T3, T2 versus T4, T3 versus T4, and T4 versus T5,* p* < 0,001 and T2 versus T5;* p* > 0,05 (NS). PEEP 0 versus PEEP 10: T1–T5; all* p* > 0,05 (NS).

For better description of atelectasis formation and ventilation shifts between nondependent and dependent lungs we calculated Impedance Ratio (IR) defined by Kunst et al. [10] as a ratio of the percent values of nondependent (ROI 1 + ROI 2) to dependent layers (ROI 3 + ROI 4) (Figure 2). Increase of IR values indicates atelectasis with ventral shift of ventilation, whereas reduction suggests more homogenous ventilation.

All parameters were recorded in 5 different time points: before induction of anaesthesia in a spontaneously breathing patient in a ramp position (T1), 5 minutes after induction and recruitment maneuver (T2), 5 minutes after insufflation of 15 mmHg pneumoperitoneum (T3), 5 minutes after moving the patient into 40° reverse Trendelenburg position (T4), and 5 minutes after exsufflation of pneumoperitoneum and moving the patient back into ramp position.

Sample size was not calculated as we did not know the exact difference size in Impedance Ratio (IR) that we should expect. However, based on previous EIT studies on ventilation distribution measured at juxtadiaphragmatic regions [11] as well as on PEEP application in normal-weight subjects [12] we assumed that minimum 32 patients would be sufficient. Shapiro-Wilk test revealed that the collected data were not normally distributed at a few individual time points in each group and consequently were all presented as box plots or median (interquartile range). For statistical comparison between different time points within each group (PEEP 0 and PEEP 10) we used Friedman test and Wilcoxon signed-rank test for post hoc analysis. Mann–Whitney test was used for PEEP 0 versus PEEP 10 comparisons.

## 3. Results

**Figure 3:**
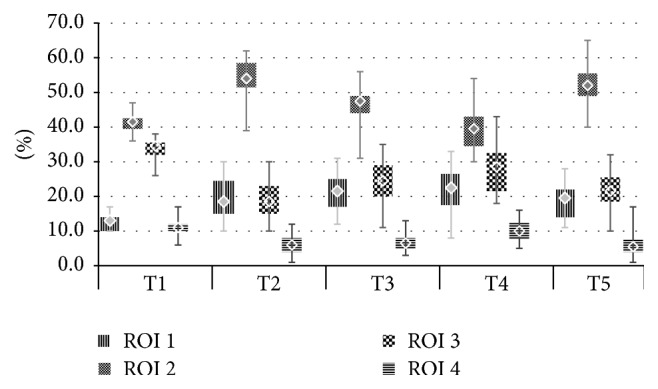
Ventilation distribution in ROI 1–ROI 4 in group PEEP 0.

**Figure 4:**
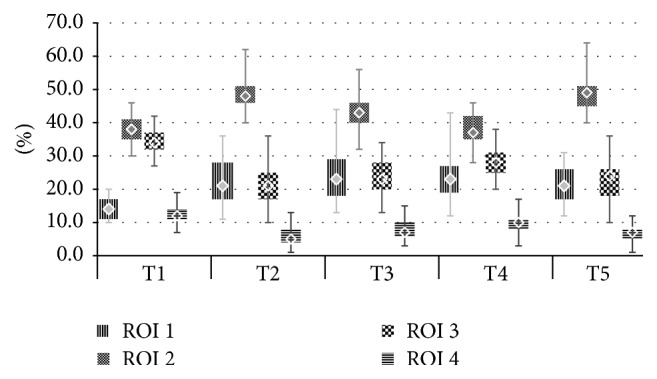
Ventilation distribution in ROI 1–ROI 4 in group PEEP 10.

8 patients (14%) were dropped from the study because high signal quality could not be obtained or was lost during anaesthesia and could not be restored before the next time point. Eventually 49 patients were included, 24 individuals in group PEEP 0 and 25 in group PEEP 10. There were no statistically significant differences between the two groups regarding initial characteristics, tidal volume settings, surgery type, and time of both anaesthesia and surgery as well as preoperative capillary blood gas (Tables 1 and 2). Comparisons between the examined time points within the two groups revealed statistically significant differences in ventilation distribution in both PEEP 0 and PEEP 10 (Figure 2). After induction of anaesthesia (T2) percent values in the nondependent layers and consequently IR ratio increased; that is, a ventral shift of ventilation was observed. After pneumoperitoneum of 15 mmHg was insufflated (T3) there was no further shift towards the nondependent lungs. On the contrary, IR ratio decreased. Reduction of IR values between T2 and T3 (Figures 3 and 4) was caused by ROI 2, 54,0 (51,5–58,5) versus 47,5 (44–49) (*p* < 0,001) and 48,0 (46–51) versus 43,0 (40–46) (*p* < 0,001) in groups PEEP 0 and PEEP 10, respectively. In ROI 1 there was no reduction of impedance variation between T2 and T3, 18,5 (15–24,5) versus 21,5 (17–25) (*p* < 0,09) and 21 (17–28) versus 23 (18–29) (*p* > 0,05) for PEEP 0 and PEEP 10, respectively.

Changing position into 40° reverse Trendelenburg (T4) resulted in additional reduction of the IR value and more homogeneous ventilation distribution. Moving back to ramp position combined with normalization of intra-abdominal pressure (T5) caused an increase in IR compared to T4. There was no significant difference in ventilation distribution measured as IR between PEEP 0 and PEEP 10 at any time point, even though absolute values of this parameter were consistently lower in PEEP 10.

**Figure 5:**
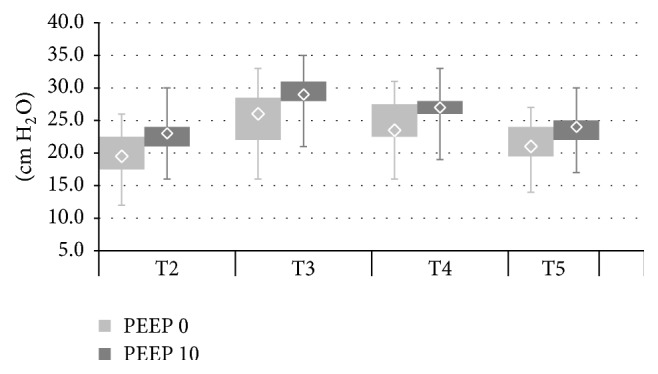
Plateau pressure (Pplat) in PEEP 0 and PEEP 10 at T2–T5. PEEP 0: T2 versus T3, T2 versus T4, T2 versus T5, T3 versus T4, and T4 versus T5; all* p* < 0,003. PEEP 10: T2 versus T3, T2 versus T4, T2 versus T5, T3 versus T4, and T4 versus T5; all* p *< 0,006. PEEP 0 versus PEEP 10: T2–T5; all* p *< 0,029.

**Figure 6:**
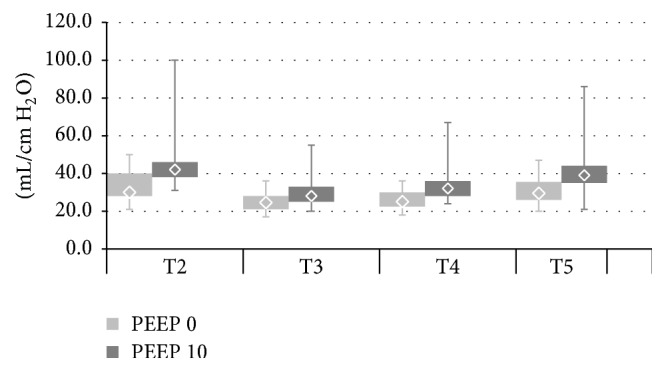
Static respiratory system compliance (Cstat) in PEEP 0 and PEEP 10 at T2–T5. PEEP 0: T2 versus T3, T2 versus T4, T2 versus T5, T3 versus T4, and T4 versus T5; all* p* < 0,018. PEEP 10: T2 versus T3, T2 versus T4, T2 versus T5, T3 versus T4, and T4 versus T5; all* p* < 0,002. PEEP 0 versus PEEP 10: T2–T5; all* p* < 0,006.

**Figure 7:**
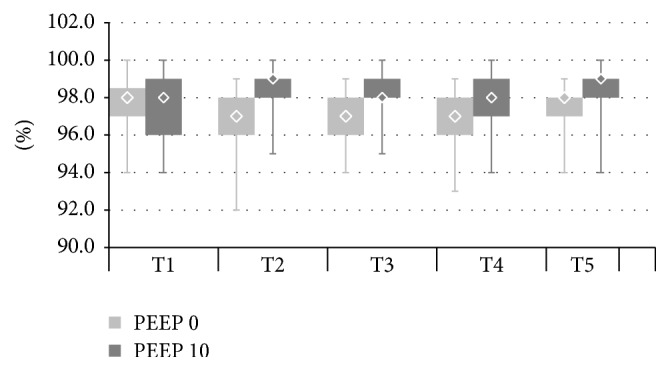
SpO_2_ in PEEP 0 and PEEP 10 at T1–T5. PEEP 0: T1 versus T2, T2 versus T3, T2 versus T4, T2 versus T5, and T3 versus T4;* p* > 0,05 (NS) and T4 versus T5;* p* < 0,04. PEEP 10: T1 versus T2, T2 versus T3, T2 versus T4, T2 versus T5, T3 versus T4, and T4 versus T5;* p* > 0,05 (NS). PEEP 0 versus PEEP 10: T2–T5; all* p* < 0,018.

The described alterations in regional ventilation distribution were accompanied by changes in the parameters of lung mechanics-plateau pressure (Pplat) and static respiratory compliance (Cresp) (Figures 5 and 6). In both PEEP 0 and PEEP 10 pneumoperitoneum (T3) increased plateau pressure and reduced respiratory compliance. Reverse Trendelenburg position led to small but statistically significant improvement in lung mechanics (Pplat reduction and Cresp increase). At T5 (back to ramp position, pneumoperitoneum resolved) Cresp increased and Pplat decreased again. Cresp and Pplat were significantly higher in the PEEP 10 compared with the PEEP 0 group throughout the whole study. SpO2 values were consistently higher in PEEP 10 from T2 to T5. Pneumoperitoneum did not lead to changes in SpO2 in either PEEP 0 or PEEP 10 (Figure 7). There were no significant differences between the examined groups regarding haemodynamic parameters (MBP, HR) (Table 4).

## 4. Discussion 

Reports on intraoperative use of EIT for ventilation assessment are scarce [8, 12–15]. Schaefer et al. [8] pointed out that EIT monitoring during abdominal surgery has significant limitations. These are loss of high impedance signal quality and close proximity to the surgical site if the belt is positioned between the 5th and 6th intercostal space. It is however just above the diaphragm that atelectasis and ventilation shifts between dependent and nondependent lungs show greatest magnitude [16].

In collaboration with the surgeons Schaefer et al. [8] placed the belt between the 2nd and 3rd intercostal space and obtained 93% of valid measurements. It should be noted that the studies by Karsten et al. [12] and He et al. [13] which were also conducted during abdominal laparoscopic surgery did not report on any technical problems even though the belt was placed between the 5th and 6th intercostal space. On the other hand Radke et al. [14] used EIT during orthopaedic surgery and Bordes et al. [15] during extraperitoneal laparoscopy for inguinal hernia repair. In both cases belt location did not interfere with the surgical site. The presented study is the first one to use EIT with a cranial belt location (3rd intercostal space) for laparoscopic surgery.

Induction of general anaesthesia (T2) (Figure 2) in the monitored area in both PEEP 0 and PEEP 10 resulted in atelectasis in the dependent lungs. The same was observed by Karsten et al. [12] and He et al. [13] at the 5th and by Schaefer et al. [8] between the 2nd and 4th intercostal space.

Insufflation of 15 mmHg pneumoperitoneum (T3) (Figure 2) in both examined groups did not lead to additional ventilation shift towards the nondependent lungs. On the contrary, the observed reduction in Impedance Ratio (IR) suggests more homogenous ventilation distribution. In the previously mentioned studies by He et al. [13] and Bordes et al. [15] pneumoperitoneum did cause more atelectasis and a ventral shift of ventilation. Karsten et al. [12] however also reported no additional ventilation shift during 15 mmHg pneumoperitoneum for laparoscopic cholecystectomy in patients ventilated without PEEP. All of these observations were made with EIT belt located at the 5th-6th intercostal space.

40° reverse Trendelenburg position (T4) promoted even ventilation distribution in the monitored lung area (further reduction of IR compared to T3). Moving the patients back to the original supine ramped position increased IR and led to a significant loss of ventilation in the dependent lungs.

Comparison between PEEP 0 and PEEP 10 groups revealed that positive end-expiratory pressure of 10 cm H_2_O preceded by a recruitment maneuver with peak inspiratory pressure of 40 cm H_2_O is insufficient to prevent atelectasis in the dependent lung regions as a result of anaesthesia in obese patients. Contrary to our study, Reinius et al. [1] found with computed tomography that in the obese a recruitment maneuver followed by PEEP of 10 cm H_2_O reduces atelectasis up to 20 minutes after induction of anaesthesia. In the aforementioned study however recruitment maneuver was performed with peak inspiratory pressure of 55 cm H_2_O. Pressure value determines the effects of recruitment which may subsequently be sustained by applying PEEP. PEEP of 10 cm H_2_O without recruitment maneuver has no recruitment potential and does not open the alveoli. The same is true for peak inspiratory pressure of 30 cm H_2_O·40 cm H_2_O proved effective in normal-weight individuals [12, 16]. On the other hand the results of our study are consistent with that of Erlandsson et al. [17] who found PEEP of 10 cm H_2_O to be insufficient to prevent alveolar collapse after general anaesthesia was induced in the morbidly obese. Prevention of atelectasis could only be achieved with PEEP as high as 15 cm H_2_O.

Parameters of lung mechanics may also be used to detect atelectasis and alveolar recruitment [18] and unlike EIT, which shows regional ventilation distribution in the monitored area, they are considered global parameters indicating changes in the whole lungs. Static respiratory system compliance (Cresp) and plateau pressure (Pplat) in the two examined groups showed typical changes described in earlier studies on respiratory mechanics during laparoscopy [19, 20] with reduced compliance and increased plateau pressure during pneumoperitoneum and the reverse after normalization of intraabdominal pressure. The only exception was that the increase in Cresp after changing position into reverse Trendelenburg turned out to be statistically significant. Sprung et al. [19] and Casati et al. [20] showed that placing patients in reverse Trendelenburg was not linked to increased compliance. This difference may have been due to 40° reverse Trendelenburg used in our study as opposed to the 20°–30° reported by most studies and the fact that Sprung et al. and Casati et al. did not use PEEP. Different modifications of reverse Trendelenburg position may be of significance. Valenza et al. [21] assessed the influence of PEEP and beach chair position (reverse Trendelenburg with the legs flexed in the hips) on respiratory mechanics. It was discovered that the latter, regardless of PEEP application, reduced respiratory system elastance, which indicates improved compliance.

Analysis of ventilation distribution and its relation to respiratory mechanics shows that alterations of ventilation distribution in the monitored area may not follow the direction of the accompanying changes of respiratory compliance. We expected that increased IR (ventilation shift towards the nondependent lungs) would be followed by a reduction in respiratory compliance as a result of atelectasis. Insufflation of pneumoperitoneum (T3) was indeed followed by a reduction of Cresp (Figure 6) but no increase in Impedance Ratio (IR) was detected. The decreased IR in the context of reduced respiratory compliance could of course be attributed to alveolar overdistension with resulting loss of ventilation in the nondependent lungs. However, this would have required reduced impedance variation in ROI 1 as this is the most ventral layer. In our study it was ROI 2 that was mostly responsible for the decreased impedance variation at T3 in the nondependent lungs (Figures 3 and 4).

Placing the patients in reverse Trendelenburg position (T4) recruited the dependent lungs and promoted homogeneous ventilation distribution in the monitored area (IR lower at T4 than at T3). This was accompanied by increased respiratory system compliance, particularly in PEEP 10. In fact ventilation at T4 was more evenly distributed than at T2 and T5, that is, before insufflation and after exsufflation of pneumoperitoneum. Respiratory system compliance at T4 however was lower than at T2 and T5 which suggests that at T4 there was more atelectasis in the lungs as a whole than at both T2 and T5.

The previously mentioned papers by Karsten et al. [7] and Bikker et al. [9] performed in intensive care patients examined the ratio of tidal volume to impedance variation using different belt locations on the chest. They demonstrated that the examined ratio may vary [7, 9] depending on the location and that there is not only ventral but also cranial shift of ventilation as a result of increased amount of atelectasis in the lungs [9]. This may suggest the influence of belt location on the obtained results of impedance variation and ventilation distribution.

Pulse oximetry (SpO_2_) values in PEEP 0 throughout the whole anaesthesia (T2–T5) were lower than in PEEP 10 (Figure 7). Comparisons within the two groups revealed that after insufflation of pneumoperitoneum there was no significant change in SpO_2_. Other researchers also suggested no SpO_2_ reduction [12] or even improved oxygenation as a result of pneumoperitoneum [22]. This is because increased intraabdominal pressure may be followed by improved ventilation to perfusion ratio due to hypoxic pulmonary vasoconstriction. Strang demonstrated in a porcine model [23] that during pneumoperitoneum perfusion moved to nondependent lung regions to a greater extent than ventilation which limited shunt of deoxygenated blood.

In conclusion, the electrical impedance tomography is valuable noninvasive clinical tool for evaluation of lung function in patients. Changes of ventilation distribution in morbidly obese patients as a result of general anaesthesia, pneumoperitoneum, and change of body position do occur at cranial lung regions and can be assessed with electrical impedance tomography. Induction of anaesthesia results in atelectasis in the dependent lungs and reverse Trendelenburg position leads to a more homogeneous ventilation distribution. After pneumoperitoneum is insufflated however alterations of ventilation distribution observed at the third intercostal space may not follow the direction of the accompanying changes of respiratory compliance. In the morbidly obese PEEP of 10 cm H_2_O improves respiratory compliance and oxygenation and should be applied during bariatric surgery but combined with a recruitment maneuver with peak inspiratory pressure of 40 cm H_2_O it is not sufficient to eliminate atelectasis caused by general anaesthesia.

## Figures and Tables

**Table 1:** Patient characteristics: median (IQR).

	Surgery (SG/LAGB)	Sex (f/m)	Age (years)	BMI (kg/m^2^)	Ta (min)	Ts (min)	VT (mL)
PEEP 0	5/19	18/6	38 (32,5–43,5)	42,8 (40,8–46,9)	52,5 (47,5–62,5)	32,5 (30–50)	550,0 (500–587,5)
PEEP 10	3/22	16/9	40 (31–47)	41,7 (38,8–46,8)	55 (50–65)	35 (30–45)	525,0 (500–575)
*p*	>0,05	>0,05	>0,05	>0,05	>0,05	>0,05	>0,05

**Table 2:** Patient characteristics: preoperative CBG: median (IQR).

	pH	pCO_2_ (mmHg)	pO_2_ (mmHg)	HCO_3_ (mmoL/L)	BE (mmoL/L)	SO_2_ (%)
PEEP 0	7,43 (7,4–7,46)	35 (31,9–36,9)	70,9 (66,6–78,5)	23,6 (21,3–24,2)	−0,2 (−1,8–0,5)	95,1 (93,2–96,1)
PEEP 10	7,42 (7,4–7,43)	36,2 (33,6–38,7)	69,5 (65,6–74,5)	23,3 (22,1–24,4)	−0,5 (−0,9–0,5)	94,5 (93,4–96,2)
*p*	>0,05	>0,05	>0,05	>0,05	>0,05	>0,05

**Table 3:** End-tidal CO_2_ (etCO_2_) and respiratory rate (RR): median (IQR).

Parameter	Group	T2	T3	T4	T5
etCO_2_ (mmHg)	PEEP 0	35,5 (35–37)	40 (38,5–41)	40,5 (39–42)	39 (38,5–41)
	PEEP 10	35 (34–36)	40 (38–41)	39 (37–41)	39 (38–40)

*p*		PEEP 0: T2 versus T3 and T4 versus T5; *p* < 0,016 and T3 versus T4; *p* > 0,05 (NS)			
		PEEP 10: T2 versus T3; *p* < 0,001, T3 versus T4, and T4 versus T5; *p* > 0,05 (NS)			
		PEEP 0 versus PEEP 10: T2, T3, and T5, *p* > 0,05 (NS) and T4; *p* < 0,046			

RR (1/min)	PEEP 0	11,5 (10–12)	13 (12–14)	13 (12–14)	12,5 (12–14)
	PEEP 10	11 (10–12)	12 (12–14)	12 (12–14)	12 (12–13)

*p*		PEEP 0: T2 versus T3 and T4 versus T5; *p* < 0,043 and T3 versus T4; *p* > 0,05 (NS) PEEP 10: T2 versus T3 and T4 versus T5; *p* < 0,012 and T3 versus T4; *p* > 0,05 (NS) PEEP 0 versus PEEP 10: T2–T5; all *p* > 0,05 (NS)			

**Table 4:** Mean blood pressure (MBP) and heart rate (HR): median (IQR).

Parameter	Group	T1	T2	T3	T4	T5
MBP (mmHg)	PEEP 0	107,5 (99,5–113)	85,5 (76,5–95)	93,5 (84,5–114,5)	78,5 (69,5–98,5)	95 (90–100)
	PEEP 10	108 (101–118)	81 (76–87)	101 (89–115)	90 (77–109)	99 (90–105)

*p*		PEEP 0: T1 versus T2, T2 versus T3, T2 versus T5, T3 versus T4, and T4 versus T5; *p *< 0,013 and T2 versus T4; *p* > 0,05 (NS)				
		PEEP 10: T1 versus T2, T2 versus T3, and T2 versus T5; all *p* < 0,002 and T2 versus T4, T3 versus T4, and T4 versus T5; *p* > 0,05 (NS)				
		PEEP 0 versus PEEP 10: T1–T5; all *p* > 0,05 (NS)				

HR (1/min)	PEEP 0	88 (81,5–96,0)	84 (78–89)	83,5 (79–905)	83,5 (75,5–96,5)	77,5 (66–80,5)
	PEEP 10	83 (80–92)	78 (72–88)	80 (71–88)	85 (75–95)	77 (68–83)

*p*		PEEP 0: T1 versus T2, T2 versus T5, and T4 versus T5; *p* < 0,049, T2 versus T3, T2 versus T4, and T3 versus T4; *p* > 0,05 (NS)				
		PEEP 10: T4 versus T5; *p* < 0,013, T1 versus T2, T2 versus T3, T2 versus T4, T2 versus T5, and T3 versus T4; *p* > 0,05 (NS)				
		PEEP 0 versus PEEP 10: T1–T5; all *p* > 0,05 (NS)				
